# Adsorption characteristics of an enteric virus-binding protein to norovirus, rotavirus and poliovirus

**DOI:** 10.1186/1472-6750-11-123

**Published:** 2011-12-16

**Authors:** Takahiro Imai, Daisuke Sano, Takayuki Miura, Satoshi Okabe, Keishi Wada, Yoshifumi Masago, Tatsuo Omura

**Affiliations:** 1 Department of Civil and Environmental Engineering, Graduate School of Engineering, Tohoku University, Aoba 6-6-06, Sendai, 980-8579, Japan; 2 Division of Environmental Engineering, Faculty of Engineering, Hokkaido University, North 13, West 8, Kita-ku, Sapporo, Hokkaido, 060-8628, Japan

## Abstract

**Background:**

Water contamination with human enteric viruses has posed human health risks all over the world. Reasonable and facile methodologies for recovering and quantifying infectious enteric viruses in environmental samples are needed to address the issues of waterborne viral infectious diseases. In this study, a bacterial protein that has a binding capability with several enteric viruses is discovered, and its binding characteristics were investigated for utilizing it as a viral adsorbent in virus recovery and detection technologies.

**Results:**

A gene of an enteric virus-binding protein (EVBP), derived from a monomer of a bacterial chaperon protein GroEL, was successfully acquired from a genomic DNA library of activated sludge microorganisms with nested PCR. Equilibrium dissociation constants between EVBP and norovirus-like particles (NoVLPs) of genotypes GI.7 and GII.4, estimated with quartz crystal microbalance method, were 240 and 210 nM, respectively. These values of equilibrium dissociation constant imply that the binding affinity between EVBP and NoVLPs is 1 to 3-log weaker than that in general antigen-antibody interactions, but about 2-log stronger than that in weak specific interactions of proteins with cations and organic polymers. The adsorptions of EVBP to norovirus, group A rotavirus and poliovirus type 1 were found to be significant in enzyme-linked immunosorbent assay. Meanwhile, the binding of native GroEL tetradecamer to viral particles was weaker than that of EVBP, presumably because of a steric hindrance. The small molecule of EVBP could have an advantage in the access to the surface of viral particles with rugged structure.

**Conclusions:**

EVBP that has a broad binding spectrum to enteric viruses was newly discovered. The broad binding characteristic of EVBP would allow us to utilize it as a novel adsorbent for detecting diverse enteric viruses in clinical and environmental samples.

## Background

Water contamination with human enteric viruses is a growing concern, because the dissemination of human enteric viruses in reservoir, river water and seawater could pose human health risks for infectious viral diseases via drinking water [1,2], recreation water [3], irrigation water [4], marine cultured products [5,6], and so on. The quantity of infectious enteric viruses in water, which has to be accurately determined for assessing risks of waterborne viral diseases, is often very difficult to analyze, because of the low concentration of enteric viruses in water environments [7], the inability of molecular detection methods to distinguish infectious and non-infectious virions [8,9], and the presence of inhibitory substances for the processes of genome extraction and amplification [10]. Reasonable and facile methodologies for recovering and quantifying infectious enteric viruses in environmental samples are needed to address the issues of waterborne viral infectious diseases.

One rationalized approach to collect infectious virions from environmental samples would be the employment of viral antigen recovery methods by using virus-specific antibodies [11]. The integrity of viral capsid structure, including antigenic sites, is prerequisite to maintain infectivity, and viral particles bound to specific antibodies might be structurally intact. However, diverse enteric viruses in *Caliciviridae*, *Picornaviridae *and *Reoviridae *have been recognized as waterborne agents [12], and antibody may be too specific to capture all possible strains of a target virus [13]. The preparation of an array of antibodies for each enteric virus of interest would be enormously costly and unrealistic. The discovery of alternative viral adsorbents, which have a broad binding spectrum to enteric viruses and ensure the infectivity of recovered virions, could be very valuable for accurately investigating health risks posed by infectious enteric viruses in water environments.

Virus-binding proteins, which were discovered from a bacterial culture derived from activated sludge [14], could be alternative viral adsorbents, because these bacterial proteins can stably interact with human viral particles [15]. Recently, norovirus-binding proteins (NoVBPs) were acquired with an affinity to a norovirus (NoV) capsid peptide [16]. It was expected that these NoVBPs, which are derived from homologues of a chaperonin protein GroEL, had a binding capability to several NoV genotypes, presumably because of the critical role of hydrophobic effect [16]. This feature of a broad binding spectrum has raised the expectation that the acquired NoVBPs might be utilized as adsorbents for the recovery and detection of multiple genotypes of NoV in environmental samples. Furthermore, the main contribution of hydrophobic effect might allow us to utilize these proteins as adsorbents for other enteric viruses, if non-specific interactions of these proteins with impurities in environmental samples are well excluded.

In this study, a gene of NoVBP was acquired from an extracted genomic DNA of activated sludge microorganisms, and the adsorption characteristics of the gene product (a candidate of enteric virus-binding protein: EVBP) to particles of enteric viruses were investigated. Values of the equilibrium dissociation constant were estimated with quarts crystal microbalance (QCM) method. Furthermore, the interaction between the EVBP candidate and enteric virus particles, including NoV genotypes GI.7, GII.3, GII.4, GII.6, group A rotavirus (RV-A) and poliovirus type 1 (Sabin) (PV1), was analyzed by enzyme-linked immunosorbent assay (ELISA). Based on these binding assays, the applicability of the gene product as EVBP was discussed.

## Results

### Production and purification of the EVBP candidate

**Figure 1:**
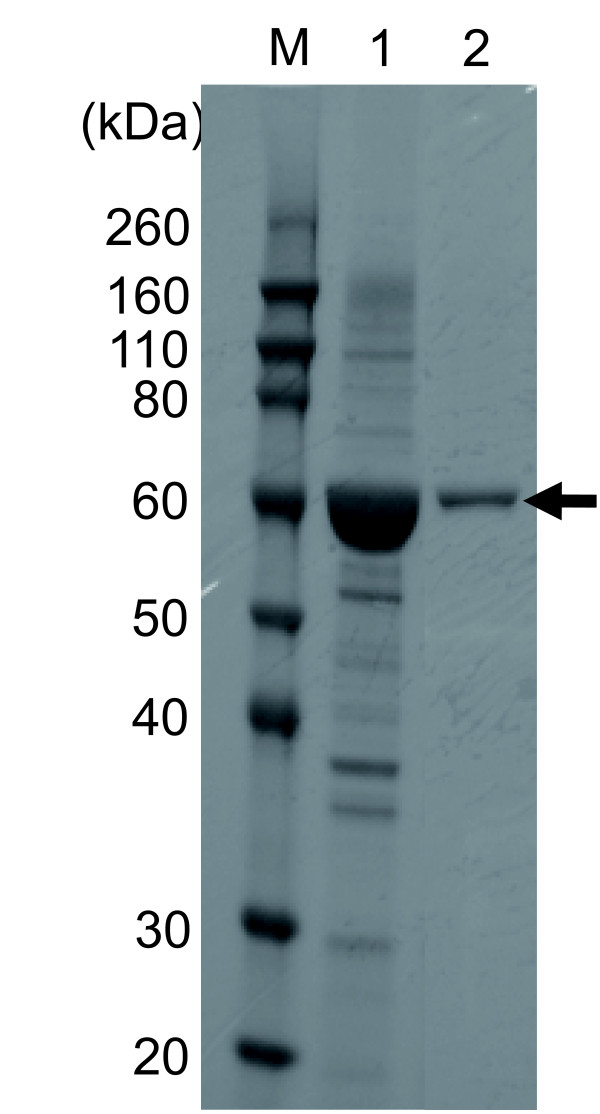
**Purification of the histidine-tagged EVBP candidate with nickel column chromatography**. Lane M, molecular marker; Lane 1, extracted soluble proteins from *E. coli*; Lane 2, proteins recovered in the elution step. Black arrow indicates the position of the histidine-tagged EVBP candidate.

A gene coding the candidate of EVBP was acquired from the extracted genomic DNA of activated sludge microorganisms with nested PCR [GenBank:AB618053]. The estimated molecular weight of the protein is 57 kDa. It was confirmed by BLAST search that the isolated gene codes a homologue of a chaperonin GroEL monomer. Figure 1 shows the SDS-PAGE profile of extracted soluble proteins from *E. coli *cells transformed by pDEST17/TI/EVBP and the EVBP candidate purified with nickel ion-immobilized affinity chromatography. Strong expression of a protein with a molecular weight of about 60 kDa was observed in the extracted soluble proteins from *E. coli *cells (Lane 1, Figure 1). This protein seemed to be histidine-tagged, because it was easily purified by nickel ion-immobilized column (Lane 2, Figure 1). These results indicate that the production and purification of the EVBP candidate were successfully performed. The recovery of the target his-tag protein as a soluble protein enormously facilitates the protein processing, because it is not necessary to purify and solubilize inclusion bodies in *E. coli *cells.

### Equilibrium dissociation constant estimated with QCM

**Figure 2:**
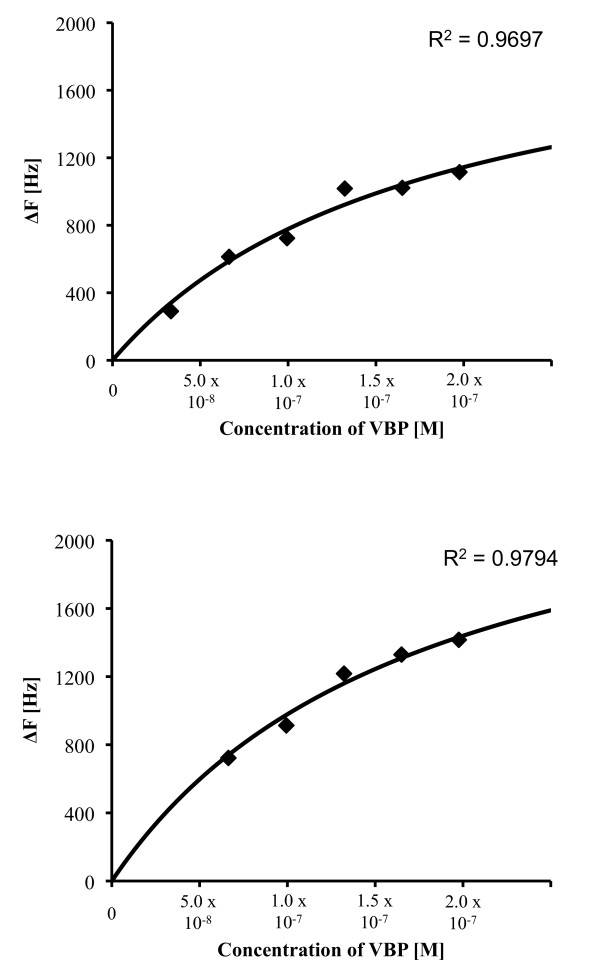
**Equilibrium adsorption isotherm of the interaction between immobilized NoVLP on a sensor chip and EVBP, acquired with the quartz crystal microbalance method**. The change of frequency (delta-F) was plotted against the concentration of EVBP added on a sensor chip where NoVLP was immobilized. A, representative delta-F spots and a regression curve for NoVLP of GI.7; B, representative delta-F spots and a regression curve for NoVLP of GII.4.

**Figure 3:**
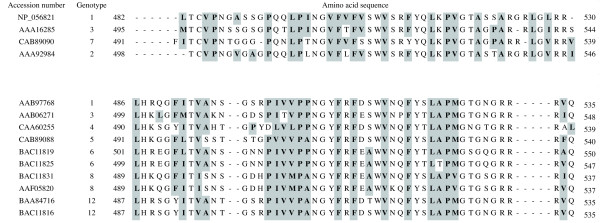
**Multiple alignments of amino acid sequences from the C-terminal region of the norovirus (NoV) capsid protein**. C-terminal regions of capsid protein from human NoV genotypes GI.1 ([GenBank:NP_056821]), GI.2 ([GenBank:AAA92984]), GI.3 ([GenBank:AAA16285]), GI.7 ([GenBank:CAB89090]), GII.1 ([GenBank:AAB97768]), GII.3 ([GenBank:AAB06271]), GII.4 ([GenBank:CAA60255]), GII.5 ([GenBank:CAB89088]), GII.6 ([GenBank:BAC11819] and [GenBank:BAC11825]), GII.8 ([GenBank:BAC11831] and [GenBank:AAF05820]), and GII.12 ([GenBank:BAA84716] and [GenBank:BAC11816]) were aligned by ClustalX 2.0.10 http://mac.softpedia.com/get/Math-Scientific/ClustalX.shtml. Hydrophobic residues were indicated by grey boxes.

Figure 2 shows the equilibrium adsorption isotherm of the interaction between immobilized NoVLP (GI.7 or GII.4) on a quartz crystal chip and the EVBP candidate, which was analyzed by QCM. The adsorption isotherm was acquired four times for each NoV genotype, and the values of the equilibrium dissociation constant in the Langmuir binding isotherm equation were estimated. As a result, the equilibrium dissociation constants of the EVBP candidate with NoV GI.7 and GII.4 were found out to be 240 nM (standard deviation (SD) = 60, n = 4) and 210 nM (SD = 50, n = 4), respectively. These values of the equilibrium dissociation constant imply that the binding between the EVBP candidate and NoVLP is 1 to 3-log weaker than that in the specific interaction between antibody and antigen, which can reach 0.1 nM [17-19]. Meanwhile, the interaction between the EVBP candidate and NoVLP is about 2-log stronger than protein adsorptions to multivalent cations and sepharose polymers, in which the dissociation constants are around several μM [20-22]. Our previous study showed that bacterial proteins with a high identity in amino acid sequence to the GroEL monomer were recovered by their affinity to C-terminal peptide originated from NoV GII.4 capsid, and the hydrophobic effect was likely to be the main factor in the interaction [16]. As indicated in Figure 3, hydrophobic amino acid residues are well conserved among NoV genotypes in genogroups I (GI) and II (GII). It would be plausible that the hydrophobic region in the C-termini of NoV capsid protein, which is exposed to outside of viral particle [23,24], is the binding site with the EVBP candidate. The gene product can be regarded as EVBP if it exhibits this level of binding affinity to other enteric viruses as well as to NoV.

### Binding assay of the EVBP candidate with enteric virus particles by ELISA

In order to confirm the binding capability of the EVBP candidate to enteric viruses, ELISA using particles of PV1, RV-A, NoV GI.7, GII3, GII.4, and GII.6 was conducted. The binding assay of a native GroEL, a tetradecamer molecule (Sigma, Japan), was also performed in order to investigate the influence of the complex formation on virus-binding ability. The EVBP candidate and GroEL complex were biotinylated prior to the binding assays, and the binding of biotinylated proteins to immobilized viral particles was detected in ELISA with avidin carrying horseradish peroxidase.

**Figure 4:**
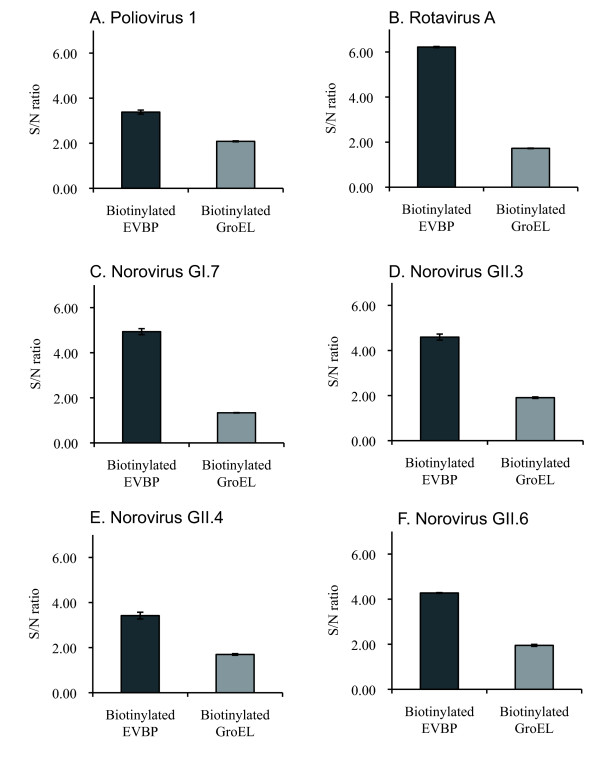
**Binding assay of the biotinylated EVBP and the native GroEL complex with enteric virus particles**. Error bars indicate the maximum and minimum values in duplicates. A, poliovirus type 1 (Sabin); B, group A rotavirus; C, NoVLP of GI.7; D, NoVLP of GII.3; E, NoVLP of GII.4; F, NoVLP of GII.6.

Figure 4 shows the result of ELISA, expressed in signal/noise (S/N) ratio. The S/N ratio larger than 1.0 means that the binding is significant, because this is the ratio of absorbance at 492 nm (A_492_) in a virus-immobilized well to that in a virus-negative (bovine serum albumin (BSA)-coated) well. Values of A_492 _in the virus-negative well were 0.18 (SD = 0.01) and 0.73 (SD = 0.07) for the biotinylated EVBP and GroEL-inoculated wells, respectively. The background signal caused by the interaction of the biotinylated GroEL with the blocking agent (BSA) was detected. Consequently, the EVBP candidate can bind with all virus particles tested (PV1, RV-A, NoV GI.7, GII.3, GII.4, and GII.6), indicating that the product of the acquired gene can be regarded as EVBP. The hydrophobic effect could play a crucial role in the binding of EVBP with PV1 and RV-A, because PV has extensive hydrophobic surface on the north rim of the canyon on the capsid surface [25], and RV has a hydrophobic apex on the exposed surface of the virion-distal end (shoulder surface) of the spike body [26]. Meanwhile, the GroEL complex also gave positive S/N ratios for all six viral particles but the values of S/N ratio were always smaller than that of EVBP. The high background signal caused by the adsorption of GroEL to BSA would be a hindrance when the GroEL is used as a viral adsorbent.

## Discussion

The present study investigated the applicability of EVBP as a viral adsorbent for diverse enteric viruses in families of *Picornaviridae*, *Caliciviridae *and *Reoviridae*, which are dominant etiological agents of gastroenteritis cases all over the world [12]. A broad binding spectrum to human enteric viruses with a sufficient binding affinity is an indispensable binding characteristic of EVBP for utilizing it as a virus adsorbent. However, too low binding specificity is not preferred, because it would cause the non-specific adsorption of impurities co-recovered with viral particles from environmental samples, which must hamper the interaction between EVBP and viral particles.

**Figure 5:**
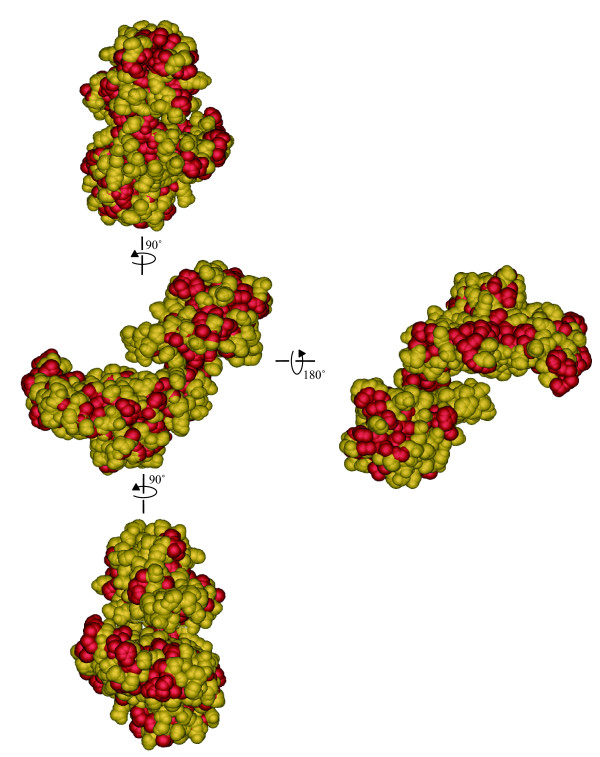
**Tertiary structure of an enteric virus-binding protein estimated with SWISS-MODEL http://swissmodel.expasy.org/**. Four lateral faces were indicated. Hydrophobic residues (alanine, glycine, isoleucine, leucine, methionine, phenylalanine, proline and valine) were turned in red (Tryptophan was not included in the EVBP sequence). The three dimensional structure was visualized with iMol http://www.pirx.com/iMol/.

**Figure 6:**
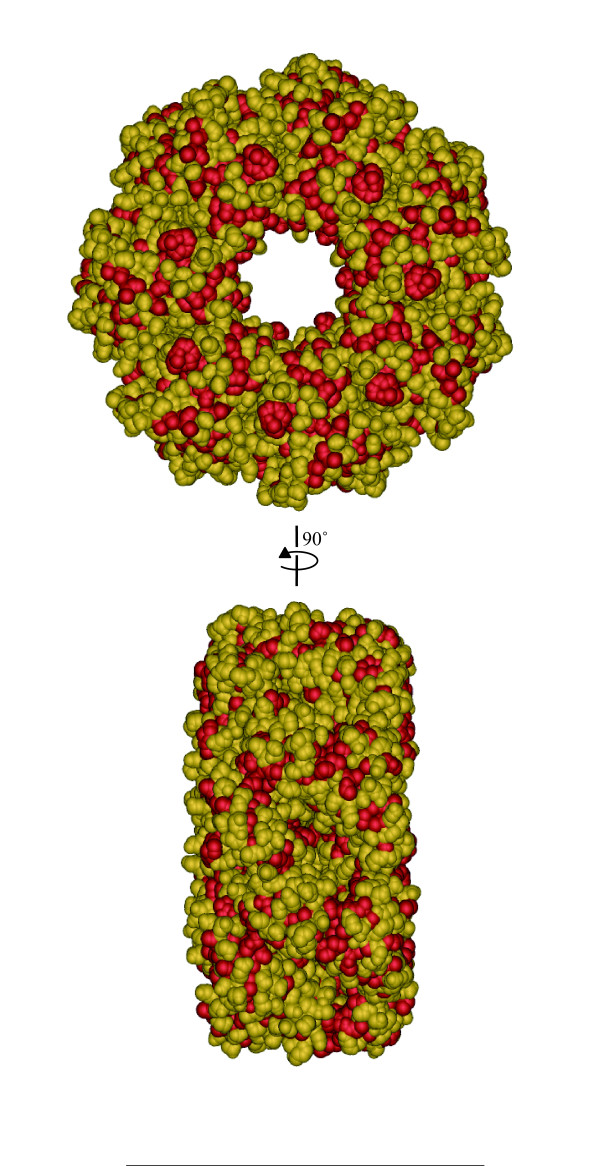
**Tertiary structure of the GroEL complex [PDB:**1SS8**]**. Two lateral faces were indicated. Hydrophobic residues (alanine, glycine, isoleucine, leucine, methionine, phenylalanine, proline, tryptophan and valine) were turned in red. The three dimensional structure was visualized with iMol http://www.pirx.com/iMol/.

A monomer of a chaperon protein GroEL was regarded as an EVBP candidate in this study. Figure 5 shows three dimensional model of EVBP, estimated by SWISS-MODEL, showing that hydrophobic residues are widely distributed on its surface. Meanwhile, the native GroEL complex, composed of 14 identical monomers [27], is involved in the folding of denatured proteins and de novo proteins just after translation [28]. GroEL can interact with denatured proteins that are exposing hydrophobic amino acid residues [29], thus having a relatively broad spectrum of binding to thousands of protein that have different amino acid sequences and conformations [30]. As shown in Figure 6, hydrophobic residues are also widely distributed on the surface of the native GroEL complex [31]. Since the GroEL complex can participate in the hydrophobic interactions [32], the difference in the binding affinity observed in Figure 4 could be attributable to the configurations in each protein. The C-terminal hydrophobic region of NoV capsid protein faces the hollows on the surface of the NoV particle [23,24], therefore, the GroEL complex could be too large to interact with the hydrophobic region. The hollow on the NoV particle surface has a depth of 40 Å and a diameter of 90 Å [33], while the GroEL complex has a height of 142 Å and a diameter of 140 Å [34]. Since the size of canyon on the capsid surface of PV is 12Å deep and 15 Å wide [35,36], the steric hindrance would be also significant when the GroEL complex interacts with the hydrophobic surface of the canyon rim of PV capsid protein. The association of the GroEL complex with the shoulder surface of VP4 spike protein of RV would be also difficult, because the height of the spike is 45 Å [37], about one third of the diameter of the GroEL complex molecule. The small molecule of GroEL monomer (up to several nm) must have an advantage in the access to the surface of viral particles with rugged structure.

Although the utilization of EVBP in the detection of enteric viruses from environmental samples was emphasized in this study, there are also possible applications of EVBP in virus detection from clinical samples, especially in the first screening for diagnostics of gastroenteritis. It would be possible to utilize EVBP as a ligand in chromatography and protein microarray techniques [38]. The display on bacterial cell surface [39] and the immobilization on carriers [40] could also facilitate the application of this protein as a viral adsorbent in clinical settings. The key aspect in the utilization of EVBP is to curb the non-specific interactions with impurities in clinical and environmental samples, because the hydrophobic effect is likely to be the main factor in the binding between viral particles and EVBP. Appropriate blocking reagents would be required when EVBP is utilized as a detection probe for enteric viruses. BSA was employed as a blocking reagent in the ELISA, and it was found that the binding of EVBP to BSA is negligible.

The binding spectrum of EVBP indicated in this study could be broader than that of histo-blood group antigens (HBGAs) specific to human norovirus [41], because other enteric viruses such as enterovirus and rotavirus are not using HBGAs as primary receptors on susceptible cells. The employment of porcine gastric mucin including HBGAs [42] could be able to capture other enteric viruses because of the presence of adhesive glycoproteins in mucin, although the non-specific adsorption of impurities also has to be overcome in the usage of mammalian gastric mucin as a viral adsorbent. Since pH, ionic strength and ion composition in surrounding water are ruling the adsorption of viral particles [43], it is important to take the measure of natural substances with the propensity to adhere to EVBP in various conditions in the further study. These efforts would offer new techniques using this novel adsorbent for detecting diverse enteric viruses from clinical and environmental samples.

## Conclusions

EVBP that has a broad binding spectrum to enteric viruses was newly discovered. EVBP is derived from a monomer of a bacterial chaperon protein GroEL, and this small hydrophobic protein molecule of EVBP could have an advantage in the access to the surface of viral particles with rugged structure. The broad binding characteristic of EVBP would allow us to utilize it as a novel adsorbent for detecting diverse enteric viruses in clinical and environmental samples.

## Methods

### Test virus and cells

Buffalo green monkey kidney (BGMK) cells and fetal rhesus monkey kidney cells (MA-104) were cultured in Eagle's minimal essential medium (MEM) with Earle's salts containing 10% (vol/vol) fetal bovine serum, 0.075% NaHCO_3_, 2 mM L-glutamine, 10 mM nonessential amino acids, 100 mg/ml penicillin, and 100 U/ml streptomycin. Cells were grown to a confluent monolayer at 37°C with 5% CO_2 _in a humidifying incubator. PV1 and RV-A was propagated in BGMK and MA-104 cells for 3 to 5 days at 37°C, respectively. PV1 was provided by Dr. Kazuyoshi Yano, Tokyo Metropolitan Institute of Public Health, Japan. RV-A was kindly supplied by Prof. Osamu Nakagomi, Graduate School of Biomedical Sciences, Nagasaki University, Japan. Virus stock was prepared by the freeze-thaw method followed by polyethylene glycol precipitation [44] and kept in 200 mL aliquots at 20°C until use. NoVLPs of genotypes GI.7, GII.3, GII.4 and GII.6 were prepared as described elsewhere [45].

### Acquisition of a gene coding a possible enteric virus-binding protein

A genomic DNA of activated sludge microorganisms was extracted as previously described [15]. Extracted genomic DNA of activated sludge microorganisms was treated with DNase-free RNase A and purified with phenol/chloroform and 100% ethanol. The target gene was acquired from the extracted genomic DNA of activated sludge microorganisms by nested PCR. Primers used in this study (Table 1) were designed based on the gene sequence of GroEL from *Aeromonas sarmonicida *(accession number: CP000644.1), because it gave 100% identity with the N-terminal amino acid sequences of NoVBPs isolated in our previous study [16]. PCR Master (Roche Diagnostics, Basel, Switzerland) was employed in all steps of DNA amplification. The 1st and nested PCR were run with 1 cycle of 95°C for 5 min, 35 cycles of 95°C for 0.5 min, 50°C for 0.5 min, and 72°C for 1 min, and 1 cycle of 72°C for 10 min. The product size of the 1st PCR is 1823, and that of the nested PCR is 1713. Results were visualized with gel electrophoresis using 1.0% agarose gels (stained with ethidium bromide) and UV light. Positive PCR bands were cut from agarose gels, and DNA was purified with GENECLEAN II KIT (Qbiogene, France). Purified products were ligated into pGEM-T Easy (Promega, WI, USA), which was used for the transformation of *E. coli *DH5 alpha (TaKaRa, Shiga, Japan). After cultivation at 37°C overnight, white colony was picked up, and the vector was extracted with Mini Prep (TaKaRa, Shiga, Japan). The sequence of the inserted PCR product was analyzed by direct sequencing of both strands of PCR product with ABI PRISM310 Genetic Analyzer (Applied Biosystems Japan Ltd., Tokyo, Japan) according to the PCR cycle sequencing big-dye terminator protocol. Based on the determined sequence, the tertiary structure was estimated with SWISS-MODEL http://swissmodel.expasy.org/[46], and visualized with iMol http://www.pirx.com/iMol/.

**Table 1:** Primers used in this study

Primer	Sequence (5' - 3')	Length	Position^a^	Product size
EVBP-SP1-F	aaccgacatcctggcgattg	20	658	1823
	
EVBP-SP1-R	caaacccagtgcacgcatac	20	2480	

EVBP-SP2-F	cgaagcgtaatccacccttt	20	679	1713
	
EVBP-SP2-R	agccgtttggtaaccaagac	20	2391	

EVBP-FW	caccatggcagctaaagaag	20	729^b^	1634^c^
	
EVBP-RV	ttacaccatgcccatgccac	20	2358	

a, position in the *Aeromonas salmonicida *chaperonin GroES and chaperonin GroEL genes, complete cds (AF030975.1); b, without 5'-cacc-3' region; c, with 5'-cacc-3' region.

### Construction of the cloning vector for EVBP candidate

A clone of the target gene was amplified by PCR with primers EVBP-FW and EVBP-RV (Table 1). EVBP-FW contains 5'-CACC-3' region, which is used for PCR Directional TOPO cloning (Invitrogen, CA, USA). PCR master was used for the DNA amplification. The PCR profile was run with 1 cycle of 95°C for 5 min, 35 cycles of 95°C for 0.5 min, 50°C for 0.5 min, and 72°C for 1 min, and 1 cycle of 72°C for 10 min. The PCR product size is 1634. PCR product was cloned with pENTR/SD/D-TOPO (Invitrogen Corp., Carlsbad, CA, USA). The cloned vector, pENTR/KW/EVBP, was used for the transformation of *E. coli *cells (One Shot TOP10 Chemically Competent Cell, Invitrogen Corp., Carlsbad, CA, USA). The target gene in pENTR/KW/EVBP was subcloned into pDEST17 (Invitrogen Corp., Carlsbad, CA, USA) with LR reaction. The pDEST17 carrying the target gene (pDEST17/TI/EVBP) was used for the transformation of *E. coli *DH5 alpha and purified as described above.

### Production of EVBP candidate with *E. coli*

Purified pDEST17/TI/EVBP was used for the transformation of *E. coli *BL21-AI (Invitrogen Corp., Carlsbad, CA, USA). A preculture containing the expression plasmid was grown overnight at 37°C in LB medium with ampicillin (50 mg/ml) and used to inoculate (1/50) LB medium. The culture was grown for 4 hrs at 37°C, and protein expression was induced by the addition of 20% L-arabinose to a final concentration of 0.5%. After overnight-growth at 37°C, cells were immediately collected by centrifugation at 9,000 xg for 10 min at 4°C. The extraction of soluble proteins was performed by BugBuster (Novagen, Darmstadt, Germany). Histidine-tagged EVBP candidate was purified with His Trap FF crude (GE Healthcare, UK). Purified EVBP candidate was desalted by dialysis against 20 mM NH_4_HCO_3 _(pH: 8.0) at 4°C for at least 12 hr, and concentrated with a vacuum and centrifugal dehydrator (CVE-100, EYELA, TOKYO RIKAKIKAI CO. Ltd., Tokyo, Japan). EVBP candidate in the pellet was suspended in 100 μl of double-autoclaved milliQ water, and processed for SDS-PAGE and silver staining as described previously [15].

### Estimation of equilibrium dissociation constant

QCM method was employed to estimate the equilibrium dissociation constant between the EVBP candidate and NoVLPs. Firstly, 50 μL of NoVLP suspension of GI.7 or GII.4 in PBS at the concentration of 10^10 ^particles/mL were inoculated on the surface of a sensor chip and left for 1 hr. NoV GII.4 is employed in the QCM test because this genotype is the most important etiological agent over the world [47]. NoV GI.7 is also detected in epidemiological studies relatively frequently among NoV GI strains [48]. The sensor chip was washed with MilliQ water and installed in QCM machine (Single-Q0500, BioLab). Then, 1 mg/ml of the EVBP candidate suspended in PBS (pH: 6.5) was inoculated on the surface of a sensor chip, and the frequency of chip was recorded. The amount of the EVBP suspension inoculated on the sensor chip was changed from 1 to 8 μl. The equilibrium dissociation constant in Langmuir binding isotherm equation was estimated with the associated analytical software Q-UP.

### Binding assay of EVBP candidate with ELISA

A 50 μl portion of virus (10^9 ^copies/well) or NoVLP (10^9 ^particles/well) suspension was added to each well of a microtiter and left for 1 hr to coat the well. Triplicate wells were used for each sample. Then, wells were washed twice with PBS and blocked with 5% BSA in PBS. After incubation at room temperature for 2 hrs, the wells were washed twice with PBS, and the biotinylated EVBP candidate in 50 μl of PBS containing 5% BSA was applied to the wells. Plates were incubated at room temperature for 1 hr and washed twice with PBS and then horseradish peroxidase (HRP)-modified streptavidin (Wako, Osaka, Japan) in 50 μl of PBS containing 5% BSA was inoculated to each well. After incubation at room temperature for 1 hr, wells were washed four times with PBS, and HRP-modified streptavidin bound was measured by coloring with o-phenylenediamine (P-7288, SIGMA CHEMICAL CO., ST. Louis, MO., USA) and H_2_O_2 _in citrate-phosphate buffer for 30 min. The coloring reaction was stopped with 2 M H_2_SO_4_. The absorbance at 492 nm (A_492_) was determined with a plate reader (Multiskan MS, Labsystems, Finland).

## Competing interests

The authors declare that they have no competing interests.

## Authors' contributions

TI carried out the protein purification and the immunoassay, participated in the sequence alignment and helped to draft the manuscript. DS conceived of the study, participated in its design and coordination and drafted the manuscript. TM carried out the molecular genetic studies and the protein binding assay. SO participated in the design of the study. KW carried out the molecular genetic studies, participated in the sequence alignment. YM participated in the design of the study. TO conceived of the study, and participated in its design. All authors read and approved the final manuscript.
